# Disseminated peritoneal coccidioidomycosis in pregnancy following fertility treatment: A case report and literature review

**DOI:** 10.1016/j.crwh.2021.e00299

**Published:** 2021-02-12

**Authors:** Armstrong Abigail, Watanabe Mika, Bhattacharya Debika, Zakhour Mae, Kroener Lindsay

**Affiliations:** a Department of Obstetrics and Gynecology, Division of Reproductive Endocrinology and Infertility, University of California Los Angeles, USA; b Department of Internal Medicine, Division of Infectious Disease, University of California Los Angeles, USA; c Department of Obstetrics and Gynecology, Division of Gynecologic Oncology, University of California Los Angeles, USA

**Keywords:** Fertility, Pregnancy, Coccidioidomycosis, Immune tolerance

## Abstract

Disseminated peritoneal coccidioidomycosis in the setting of early pregnancy after fertility treatment is rare and can present as a diagnostic challenge. A 39-year-old underwent ovarian stimulation with clomiphene citrate followed by HCG trigger and intrauterine insemination. She developed persistent abdominal pain, ascites and episodes of fever in early pregnancy, and eventually underwent a diagnostic laparoscopy for worsening clinical presentation. Operative findings were notable for peritoneal studding, infracolic omentum inflammation, bowel adhesions to the abdominal wall and normal-appearing uterus and adnexa. The pathology results indicated peritoneal *Coccidioides immitis* infection. Hormonal changes associated with fertility treatment and immune tolerance in pregnancy may increase the risk for disseminated peritoneal coccidioidomycosis. A high index of suspicion and a multidisciplinary team are important for the diagnostic workup and treatment plan of disseminated peritoneal coccidioidomycosis.

## Highlights

• We report a rare case of isolated peritoneal coccidioidomycosis in early pregnancy presenting acutely after infertility treatment.
• A high index of suspicion and multi-disciplinary team are important for the diagnostic workup and treatment plan of disseminated peritoneal coccidioidomycosis.

## 1 Introduction

Coccidioidomycosis is a fungal infection that results primarily in pulmonary disease and is endemic to the Southwest United States, Mexico and South America [1]. Extrapulmonary dissemination occurs in 1% of cases, usually in patients with risk factors like male sex, African ancestry, immunosuppression and pregnancy [[2], [3], [4]]. Prior data have shown that disseminated disease occurs in 23% of pregnant patients in the first trimester [5]. Extrapulmonary disease occurs most commonly in soft tissue, skin, joints and brain [6]. In contrast, peritoneum dissemination is rare, occurring through hematogenous spread or swallowing pulmonary secretions [7]. Disseminated cocciodiodes, like tuberculosis, is a great mimicker and can be challenging to diagnose. For example, early infection may present with persistently negative antibodies, especially in patients who are immunosuppressed or immune tolerant, as observed in pregnant women [3]. Additionally, there is a wide sensitivity and specificity of the enzyme immunoassay test, depending on lab assay [3].

To our knowledge, there are no reported cases of disseminated peritoneal coccidioidomycosis in the setting of early pregnancy following fertility treatment. We present the case of a 39-year-old woman with an early pregnancy resulting from ovarian stimulation with clomiphene citrate and intrauterine insemination who developed episodes of fever, ascites and abdominal pain ultimately requiring an exploratory laparotomy leading to a diagnosis of disseminated peritoneal *Coccidioides immitis*.

## 2 Case Presentation

A 39-year-old woman presented to an infertility clinic. She was diagnosed with unexplained secondary infertility associated with male factor. Her workup showed normal day 3 laboratory results (FSH 7.0 mIU/mL, estradiol 35 pg/mL), TSH 2.8 mcIU/mL, normal hysterosalpingogram, antral follicle count of 32 and semen analysis with mild oligoteratospermia. The couple had negative infectious disease screening, including hepatitis B and C, HIV, syphilis and HTLV 1–2. She underwent her first clomiphene citrate 50 mg cycle and developed two dominant follicles. Ovulation was triggered using 250 μg of HCG subcutaneously. She underwent an uncomplicated intrauterine insemination with 24 million fresh, motile washed sperm.

**Fig. 1:**
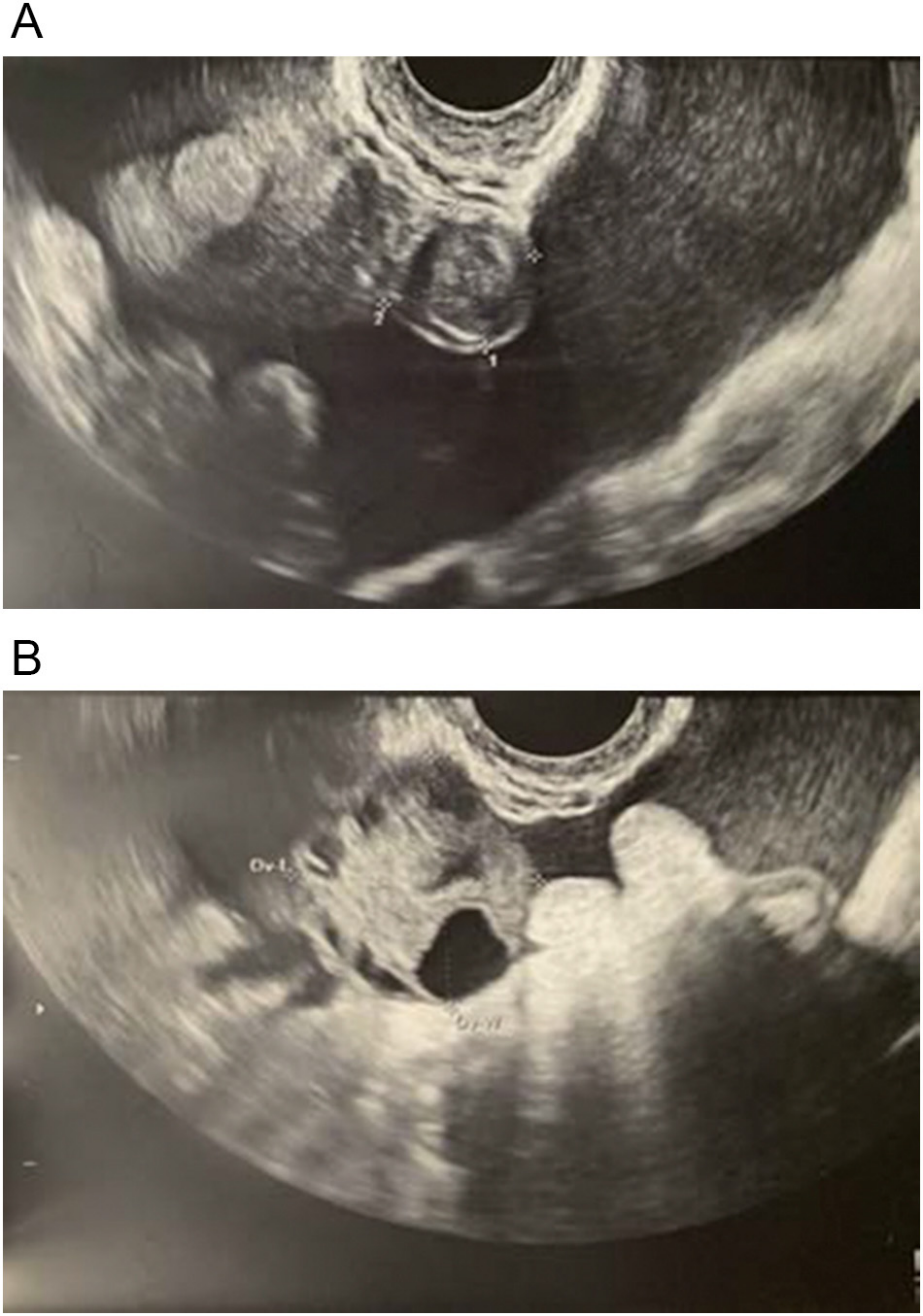
A. Large amount of free fluid in pouch of Douglas. B. Left ovary with corpus lutea.

She began experiencing intermittent fevers and abdominal pain 8 days after insemination. She was seen by her primary care physician, who noted normal temperature, mild tenderness on exam and negative pregnancy test. She was counseled that her symptoms may be secondary to early pregnancy or mild gastritis. Three days later, she re-presented with a mild leukocytosis, serum beta-hCG 84 mIU/ml and normal electrolytes. Pelvic ultrasound showed moderate free fluid with echogenic debris, two left ovarian corpus lutea and an unremarkable appendix (Fig. 1). Consistent with early gestation, no pregnancy was identified, and suspected diagnosis was ruptured hemorrhagic cyst. However, her symptoms continued to worsen, with increasing ascites and distension, persistent fevers, mild increase in liver enzymes (AST 96 U/L, ALT 93 U/L]) and leukocytosis (15 K/uL), and she was sent to the emergency room at 4 weeks 1 day.

She had her first documented fever (38.2 °C), up-trending leukocytosis (17 K/uL), mild increase in liver enzymes (AST 50 U/L, ALT 80 U/L), beta-hCG of 127 mIU/ml, normal electrolytes and negative blood cultures. Abdominal ultrasound showed free fluid throughout the abdomen, thickened endometrial lining and a normal appendix. In addition to abdominal distension, the patient developed diarrhea and nausea. Given early gestation and goal to avoid CT radiation, a non-contrast MRI was performed, which showed significant free fluid, two ovarian cysts, likely corpus lutea, and inability to visualize the appendix. There was low suspicion for ovarian hyperstimulation syndrome given clomiphene citrate use for stimulation, two dominant follicles at trigger, normal electrolytes and absence of enlarged ovaries.

She was admitted for IV antibiotics for suspected bacterial peritonitis. Infectious disease was consulted. Ceftriaxone, flagyl and vancomycin were used for genitourinary coverage given fertility treatments. The patient was originally from Tanzania, which made the team suspicious for tuberculosis despite lack of clinical history and x-ray showing small pleural effusions.

By hospital day (HD) 3, she was clinically worsening, with fevers (39.4 °C), rising leukocytosis (23 K/uL) and abdominal distention. CT of the abdomen with contrast showed moderate ascites, omental nodularity and caking, not previously seen on MRI, concerning for malignancy or non-malignant causes, including tuberculosis. Gynecology oncology was consulted and the tumor markers CA-125, CA19-9, CEA, CA 15-3, CA 27.29, AFP were normal. Coccidioidomycosis IgM, IgG, and complement fixation titers were negative and <1:2 respectively. In terms of her pregnancy of unknown location, beta-HCGs were abnormally rising and trended every 48 h (126 mIU/ml, 180 mIU/ml, 395 mIU/ml).

On HD 5, paracentesis drained 800 l of fluid and interventional radiology-guided omental biopsies resulted in fat necrosis and granulomatous inflammation. Both peritoneal fluid and tissue biopsies were negative for neoplastic cells, CEA, acid-fast bacilli, fungal, bacterial, non-tuberculosis mycobacterium DNA and *Mycobacterium tuberculosis* (MTB) PCR. The only pertinent test was an indeterminate serum quantiferon gold; however, peritoneal fluid adenosine deaminase and negative MTB PCR were not consistent with tuberculosis. With progressively worsening symptoms and lack of diagnosis, the decision was made to proceed with laparoscopy for tissue diagnosis. Given abnormally rising beta-HCGs, pregnancy was likely non-viable, thus she elected for termination.

**Fig. 2:**
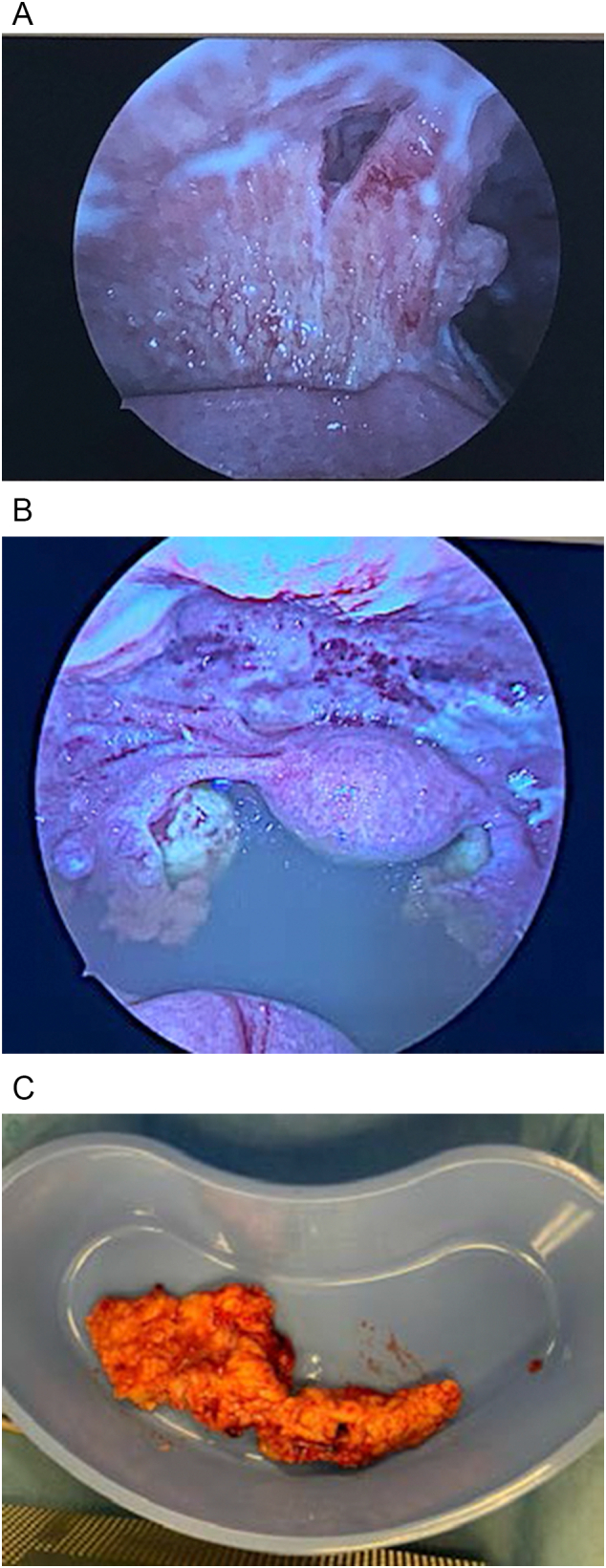
A. Bowel attached to abdominal wall upon entry. B. Laparoscopic view of uterus, adnexa and ascites. C. “Caked omentum”, pathologic specimen.

She underwent a diagnostic laparoscopy, omentectomy, lysis of adhesions and suction curettage on HD 8 with gynecology oncology, given CT results concerning for malignancy. Laparoscopy was converted to mini laparotomy due to significant adhesions and inability to obtain adequate biopsies for tissue diagnosis. Operative findings included extensive studding of peritoneum, infracolic omentum inflammation, bowel adhesions to abdominal wall, normal-appearing uterus and adnexa with 3.5 l of ascites (Fig. 2). Curettage showed decidualized endometrium without chorionic villi or coccidiodes. Beta-HCG down-trended to zero post-operatively consistent with successful surgical abortion.

Post-operatively her fevers continued despite antibiotics. Surgical omental biopsy cultures resulted as *Coccidioides immitis* on postoperative day 11. Her only notable travel history was to Palm Springs, California, and visiting construction sites in the past two years, locations known to harbor coccidioidomycosis. After significant improvement on oral antifungal therapy, she was discharged on HD 15 with fluconazole 400 mg daily. She was followed by the multidisciplinary team as an outpatient. Coccidioides antibody complement fixation (titer 1:4) turned positive one month after her admission date.

## 3 Discussion

This is a unique case describing disseminated peritoneal coccidioidomycosis in a newly pregnant patient following fertility treatment with insemination. Peritoneal coccidioidomycosis is a challenging diagnosis as there are only 34 published cases [8]. Most patients with isolated peritoneal involvement and negative serology titers are immunosuppressed patients [3,9]. It is therefore possible that our pregnant patient had falsely negative titers due to the immune tolerance of pregnancy.

Most patients with disseminated coccidioidomycosis present gradually with abdominal distension and ascites occurring months to years after infection [2]. In our patient's case, it is possible that the immune-tolerance effects of pregnancy or the hormonal effects of ovarian stimulation may have contributed to her acute presentation. Prior data report pregnant women experience disseminated disease 100 times more frequently [2,6].

Our case highlights a possible association between hormonal changes with fertility treatment, immune tolerance in pregnancy, and risk for peritoneal coccidioidomycosis. Studies show that elevated serum levels of 17-beta-estradiol and progesterone in vitro increase coccidioidomycosis growth, contributing to dissemination [10]. Our patient received clomiphene citrate and was newly pregnant, thereby raising estradiol and progesterone levels. From an immunologic standpoint, pregnancy is a state of decreased cell-mediated immunity, which explains why coccidioidomycosis disseminates more commonly in pregnancy [4,11]. Evidence shows that pregnancy increases the risk of dissemination in proportion to gestational age, with greatest risk in the third trimester, thus our patient's presentation in early gestation was unusual [4,11]. In addition to pregnancy and presumably elevated hormones levels, African ancestry is another risk factor for disseminated disease in our case [11].

The timing and source of initial infection in our patient is unclear. Coccidioidomycosis was likely contracted years prior to presentation through environmental exposure. Even in dissemination, the usual source of initial infection is the pulmonary tract [1]. It is unusual for disseminated disease to involve the female genital tract, thus the insemination procedure or specimen are unlikely to be a cause for initial infection [12]. In concordance, endometrial tissue was negative for coccidioidomycosis. In the only other report of disseminated coccidioidomycosis in a woman who underwent ovarian stimulation with clomiphene citrate and insemination, presentation was several years after fertility treatment [10].

There is limited evidence on how to treat isolated peritoneal coccidioidomycosis. Management is typically fluconazole 400 mg daily for 6–12 months with duration guided by clinical, laboratory, and radiographic response [3]. Unlike other sites of dissemination, mortality is rare with isolated peritoneal coccidioidomycosis [13]. Going forward, our patient may have a small but increased risk of disease reactivation in future pregnancies. There is a lack of literature guiding reproductive patients for timing and risk of future conception [6,14]. According to the Infectious Disease Society of America guidelines, risk of reactivation in pregnancy is low in women who have completed therapy [3]. Once pregnant, the current recommendation is coccidioidal serologic testing every 6–12 weeks [3].

## 4 Conclusion

We believe this is the only published case of isolated peritoneal coccidioidomycosis in early pregnancy. The diagnosis of peritoneal coccidioidomycosis was particularly challenging given our patient's negative infectious workup and granulomatous tissue on initial omental biopsy. This case demonstrates the importance of a multidisciplinary team. Our patient was originally from Tanzania, which prompted us to consider tuberculosis as the etiology of her disease. Ultimately, local factors like living in California, rather than global factors, were the cause of her disease.

## References

[1] Crum N.F., Lederman E.R., Stafford C.M., Parrish J.S., Wallace M.R. (2004). Coccidioidomycosis: a descriptive survey of a reemerging disease. Clinical characteristics and current controversies. Medicine (Baltimore).

[2] Phillips P., Ford B. (2000). Peritoneal coccidioidomycosis: case report and review. Clin. Infect. Dis..

[3] Galgiani J.N., Ampel N.M., Blair J.E. (2016). Infectious Diseases Society of America [IDSA] clinical practice guideline for the treatment of coccidioidomycosis. Clin. Infect. Dis..

[4] Drutz D.J., Huppert M. (1983). Coccidioidomycosis: factors affecting the host-parasite interaction. J. Infect. Dis..

[5] Hooper Jody E., Lu Qun, Pepkowitz Samuel H. (1 April 2007). Disseminated Coccidioidomycosis in pregnancy. Arch. Pathol. Lab. Med..

[6] Labuschagne H., Burns C., Martinez S. (2016). Coccidioidomycosis in pregnancy: case report and literature review of associated placental lesions. Case Rep. Womens Health.

[7] Crum-cianflone N.F., Truett A.A., Teneza-mora N. (2006). Unusual presentations of coccidioidomycosis: a case series and review of the literature. Medicine (Baltimore).

[8] Storage T.R., Segal J., Brown J. (2015). Peritoneal coccidioidomycosis: a rare case report and review of the literature. J. Gastrointestin Liver Dis..

[9] Fish D.G., Ampel N.M., Galgiani J.N. (1990). Coccidioidomycosis during human immunodeficiency virus infection. A review of 77 patients. Medicine (Baltimore).

[10] Bæk O., Astvad K., Serizawa R., Wheat L.J., Brenøe P.T., Hansen A.E. (2017). Peritoneal and genital coccidioidomycosis in an otherwise healthy Danish female: a case report. BMC Infect. Dis..

[11] Spinello I.M., Johnson R.H., Baqi S. (2007). Coccidioidomycosis and pregnancy: a review. Ann. N. Y. Acad. Sci..

[12] Smith G., Hoover S., Sobonya R., Klotz S.A. (2011). Abdominal and pelvic coccidioidomycosis. Am J Med Sci.

[13] Ellis M.W., Dooley D.P., Sundborg M.J., Joiner L.L., Kost E.R. (2004). Coccidioidomycosis mimicking ovarian cancer. Obstet. Gynecol..

[14] Walk Walker M.P., Brody C.Z., Resnik R. (1992). Reactivation of coccidioidomycosis in pregnancy. Obstet. Gynecol..

